# Antiepileptic Drug-Induced Drug Reaction with Eosinophilia and Systemic Symptoms (DRESS): A Case Series

**DOI:** 10.7759/cureus.104465

**Published:** 2026-03-01

**Authors:** Mohamed Mohaideen, Muthu Meera, Ilavendiran Sekar

**Affiliations:** 1 Pharmacology, Velammal Medical College Hospital and Research Institute, Madurai, IND; 2 Dermatology, Velammal Medical College Hospital and Research Institute, Madurai, IND

**Keywords:** anti-epileptics, pharmacovigilance, scar to phenytoin, s: dress syndrome, severe cutaneous adverse drugs reactions

## Abstract

Introduction/Background: Drug Reaction with Eosinophilia and Systemic Symptoms (DRESS) is a severe cutaneous adverse drug reaction with multisystem involvement frequently triggered by aromatic antiepileptic drugs (AED) like phenytoin, carbamazepine, etc. The pathogenesis is due to reactive metabolites and the immune reaction of delayed-type hypersensitivity. This case series addresses the key clinical presentation with pharmacological and pharmacovigilance aspects of the DRESS syndrome.

Methodology: This case series describes seven cases of AED-induced DRESS syndrome, assessed with Registry of Severe Cutaneous Adverse Reactions (RegiSCAR) criteria from a tertiary care teaching hospital in Tamil Nadu. Clinical presentation, laboratory parameters, causality assessment (WHO-UMC, Naranjo, Liverpool), preventability assessment (Modified Schumock Thornton), and severity grading (Hartwig-Siegel scale) were recorded. All assessment tools used are open access and properly cited.

Results: Phenytoin was the most common causative agent, followed by oxcarbazepine, valproate, and levetiracetam. Clinical presentation varied from five to 28 days of drug exposure. All cases were diagnosed to have *definite DRESS*. The majority of patients exhibited eosinophilia and systemic involvement in the form of hepatotoxicity. Corticosteroid therapy led to clinical resolution with hospital stays ranging from 10 to 35 days.

Conclusions: AED-induced DRESS poses a significant pharmacological challenge due to its unpredictable immunopathogenesis and genetic predisposition. Pharmacovigilance strategies and early clinical recognition by recognizing subtle laboratory parameters are essential to mitigate potential risks. Future research should focus on identifying biomarkers, optimizing drug selection, and implementing structured monitoring in high-risk populations.

## Introduction

Drug Reaction with Eosinophilia and Systemic Symptoms (DRESS) is a rare but severe cutaneous adverse reaction (SCAR) characterized by a long latency period of two to eight weeks and a prolonged clinical course that may persist even after withdrawal of the offending drug [1]. It occurs as a severe, potentially life-threatening illness with fever, rash, lymphadenopathy, hematological abnormalities, and organ involvement, mostly the liver, followed by the kidney, lungs, and rarely the heart [2]. The estimated incidence in the general population is more than 1 case per 10,000 exposures to medications [3]. Among the main triggers, antiepileptic drugs (AEDs) are frequently implicated, with aromatic AEDs historically being the most common culprits [4]. Aromatic AEDs are those AEDs that contain an aromatic benzene ring structure. They are commonly associated with hypersensitivity reactions, including SCAR. Examples include phenytoin, carbamazepine, etc. However, non-aromatic AEDs are increasingly being recognized as potential triggers [5].

The pathogenesis of DRESS involves a complex interplay of drug metabolism, immune activation, viral reactivation, and genetic susceptibility [6]. It is classified as a delayed type IV hypersensitivity reaction, driven by drug-specific T-cell activation [7]. Pharmacogenomic studies have identified strong associations with specific human leukocyte antigen (HLA) alleles, such as HLA-B*15:02 and HLA-A*31:01, particularly for carbamazepine-induced DRESS [8]. Additionally, reactivation of human herpesviruses (HHV-6, HHV-7, Epstein-Barr virus (EBV)) is observed in a significant subset of cases, suggesting a viral-immune interplay in disease pathogenesis [9].

The diagnosis remains challenging due to heterogeneous presentation. The RegiSCAR criteria, developed by the European Registry of Severe Cutaneous Adverse Reactions, are widely used for case definition and grading [10]. Other diagnostic tools include the Japanese consensus criteria and the Bocquet criteria, though each has limitations [11,12]. Early recognition and prompt withdrawal of the offending drug are critical in reducing morbidity and mortality [13]. Despite improved awareness, DRESS continues to pose diagnostic and therapeutic challenges, underscoring the need for heightened pharmacovigilance, especially in high-risk populations [14,15]. This case series aims to describe the clinical and pharmacological profile of AED-induced DRESS, highlighting diagnostic approaches, causality assessment, and pharmacovigilance implications in a tertiary care setting in South India.

## Materials and methods

Methodology

This study is a retrospective case series assessing patients who developed DRESS due to antiepileptics. The study was conducted at a tertiary care teaching hospital in Tamil Nadu, South India, from September 2024 to May 2025. The cases were identified from the Departments of Neurology and Dermatology. Included cases met the following criteria: (1) a RegiSCAR minimum score of 5 and were prioritized in descending order of score; and (2) a definitive temporal association (chronological evidence) between antiepileptic intake and the development of symptoms. Patients with active viral infection or alternative dermatological diagnoses were excluded. After obtaining informed consent, data on drug exposure, clinical presentation, and laboratory parameters were collected. Diagnosis was established based on the RegiSCAR scoring system [10]. All therapy information was diligently documented, along with the final outcomes, including resolution of clinical symptoms and normalization of laboratory parameters.

The standard causality assessment was performed using the WHO-UMC criteria [16]. To improve scientific rigor and reduce bias, we evaluated the cases using additional causality assessment scales, including the Naranjo algorithm [17] and the Liverpool ADR Causality Assessment Tool [18]. From a pharmacovigilance perspective, ADR preventability was assessed using the modified Schumock-Thornton scale [19], and severity was graded using the Hartwig-Siegel scale [20]. All tools are open access and were used with appropriate citation. No special permissions were required. Interrater reliability was assessed in consultation with the concerned faculty members; the Liverpool and Naranjo scales showed equivocal reproducibility compared with the WHO-UMC scale. All laboratory parameters were recorded at baseline at the time of DRESS diagnosis. All cases were reported to the Pharmacovigilance Programme of India (PvPI) through the designated Adverse Drug Reaction Monitoring Centre of our institute via VigiFlow.

This case series was reviewed and approved by the Institutional Ethics Committee of Velammal Medical College Hospital and Research Institute (Approval No. VMCIEC/087/2026). Patient confidentiality was preserved in accordance with the Declaration of Helsinki, and all data were de-identified for reporting.

## Results

Case presentation

The demographic characteristics of the seven included cases are summarized in Table 1. Baseline laboratory parameters, including hematological parameters, are presented in Table 2. Causality and severity assessments, along with the RegiSCAR scoring system for each case, are detailed in Table 3.

**Table 1:** Demographic details of cases.

Variable	Case 1	Case 2	Case 3	Case 4	Case 5	Case 6	Case 7	Measure of central tendency
Age (in years)	74	45	50	40	64	28	45	45 (Median)
Sex	Female	Female	Male	Male	Male	Female	Male	4:3 (Male:Female)
Latency period of onset (days)	28	5	21	14	21	10	28	21 (Median)
Offending drug	Levetiracetam	Phenytoin	Valproate	Phenytoin	Phenytoin	Oxcarbazepine	Phenytoin	Phenytoin, 4 (57.1%)
								Valproate, 1 (14.2%)
								Oxcarbazepine, 1 (14.2%)
								Levetiracetam, 1 (14.2%)
Organ involvement	Liver, lungs	Liver	Liver	Liver	Liver, Renal	Liver	Liver	Liver, 7 (100%)
								Lungs, 1 (14.2%)
								Renal, 1 (14.2%)

**Table 2:** Laboratory parameters of cases. Creatinine is reported in milligrams per deciliter (mg/dL). AEC, absolute eosinophil count (cells per microliter); ALT, alanine aminotransferase (international units per liter, IU/L); AST, aspartate aminotransferase (IU/L); ALP, alkaline phosphatase (IU/L); ULN, upper limit of normal

Variable	Case 1	Case 2	Case 3	Case 4	Case 5	Case 6	Case 7	Lab normal
AEC (cells/microliter)	916	1,745	846	1,565	1,632	1,911	1,801	35-350
ALT (IU/L)	240	89	164	126	81	205	187	0-35
AST (IU/L)	216	76	176	98	76	196	176	0-35
ALP (IU/L)	192	165	76	65	67	100	110	30-120
Creatinine (mg/dL)	1.1	0.8	0.6	0.8	1.7	0.9	1	0.7-1.4

**Table 3:** Assessment scores of each case. RegiSCAR, Registry of Severe Cutaneous Adverse Reactions

Case number	RegiSCAR	Modified Schumock-Thornton	Hartwig Severity	WHO Causality	Naranjo Scale	Liverpool Causality
1	5/7	Non-preventable	Level 5	Possible	Possible (4)	Possible
2	6/7	Non-preventable	Level 5	Certain	Probable (8)	Probable
3	5/7	Non-preventable	Level 5	Probable	Probable (7)	Possible
4	5/7	Non-preventable	Level 4b	Probable	Probable (7)	Possible
5	5/7	Non-preventable	Level 5	Probable	Probable (6)	Possible
6	6/7	Non-preventable	Level 4b	Probable	Probable (7)	Possible
7	5/7	Non-preventable	Level 4b	Probable	Probable (8)	Probable

Case 1

**Figure 1:**
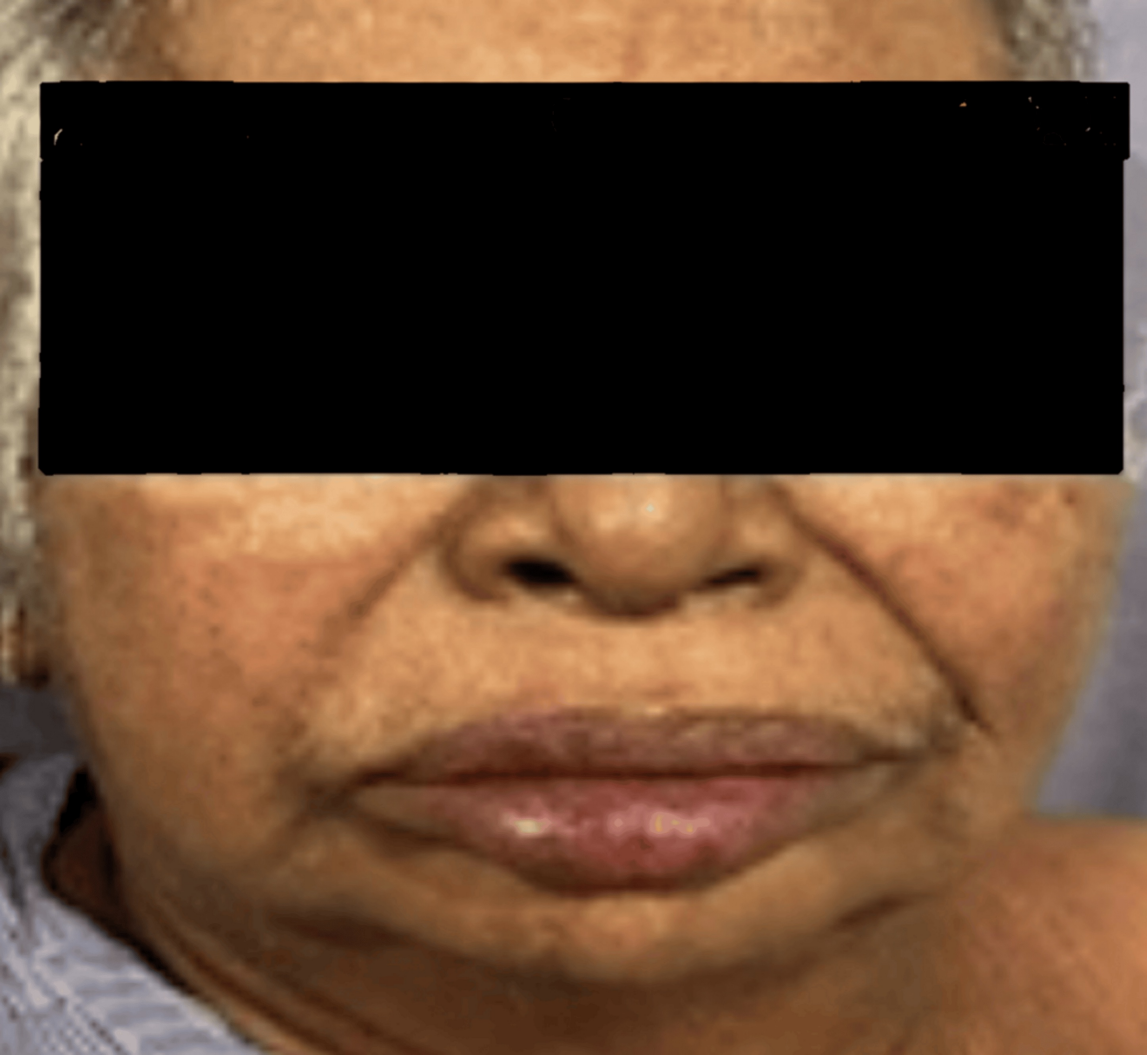
Case 1: Facial involvement with diffuse erythema and edema.

**Figure 2:**
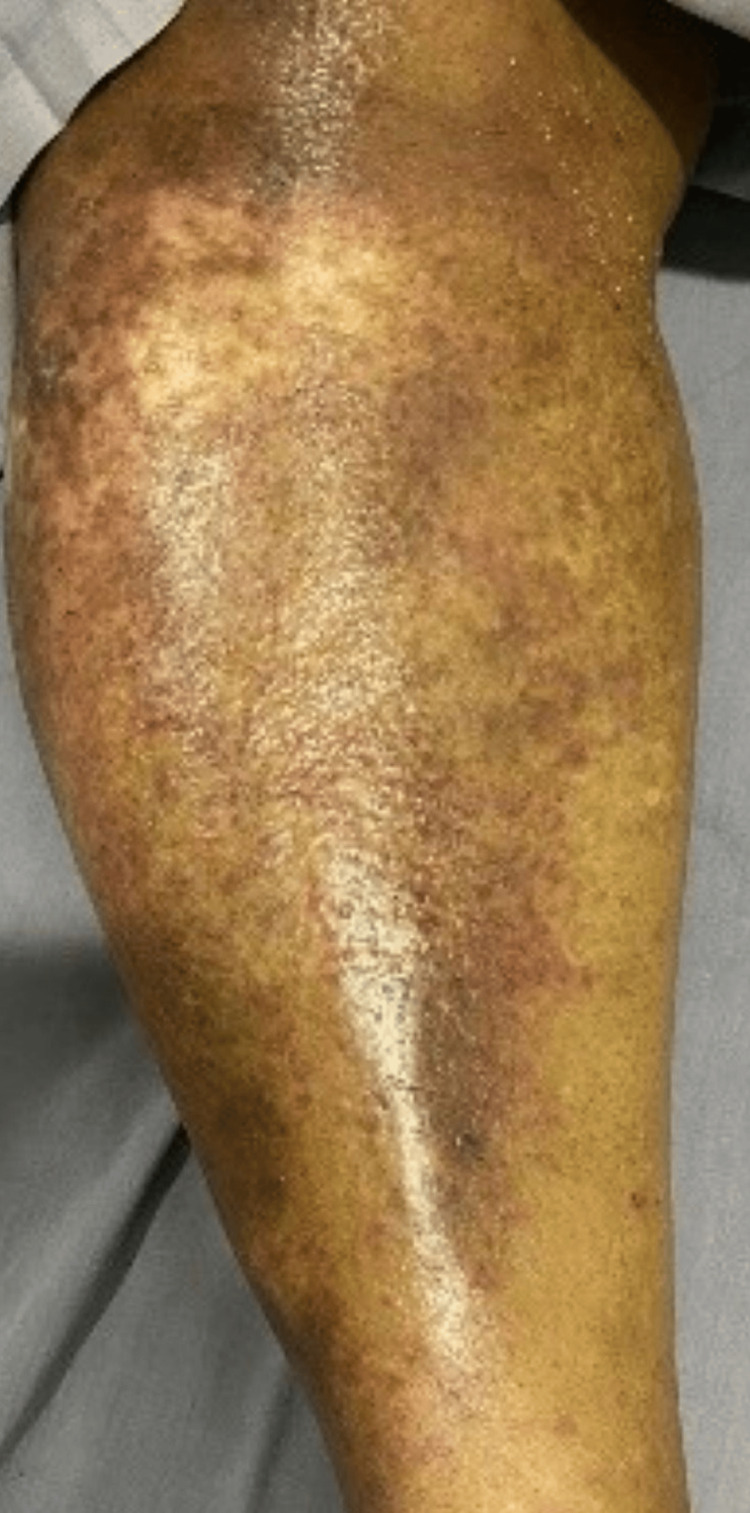
Case 1: Maculopapular rash over the lower limb.

A 74-year-old female with history of diabetes, hypothyroidism, coronary artery disease, and post aortic valve replacement, recently admitted and discharged for decompensated liver disease and hepatic encephalopathy, was started on levetiracetam 500 mg once daily. After four weeks, she presented with itching all over the body, fever, maculopapular rash, facial puffiness (Figures 1-2), swelling of both limbs with exanthema, cough and dyspnea for three days. Laboratory tests showed eosinophilia (18%) and lymphocytosis with elevated aspartate aminotransferase (AST) and alanine aminotransferase (ALT). Chest X-ray revealed patchy infiltration. Echocardiography was normal. On day 3, she developed disorientation and slurred speech; MRI showed chronic lacunar infarcts and senile atrophy. She met the criteria for *definite DRESS* by RegiSCAR. Her levetiracetam was withdrawn. She was treated with intravenous dexamethasone 8 mg once daily for five days, switched to oral prednisolone 20 mg that was gradually tapered over the next 28 days with supportive care, leading to gradual resolution. She had a hospital stay of 17 days.

Case 2

A 45-year-old female with prior glioblastoma multiforme had been on phenytoin 200 mg once daily for two years and developed DRESS, was switched to levetiracetam (500 mg once daily). Fifteen days before admission, she accidentally re-exposed herself to leftover phenytoin, and within five days developed a maculopapular rash involving all limbs, accompanied by fever, exfoliation, buccal mucosal patches, and bilateral pedal edema. Lab tests showed eosinophilia (26%), AST/ALT 3× upper limit of normal (ULN) and ALP 5× ULN. Phenytoin was stopped. Treatment with intravenous dexamethasone (8 mg daily for five days), topical medications such as mometasone, and supportive care led to resolution within 16 days.

Case 3

**Figure 3:**
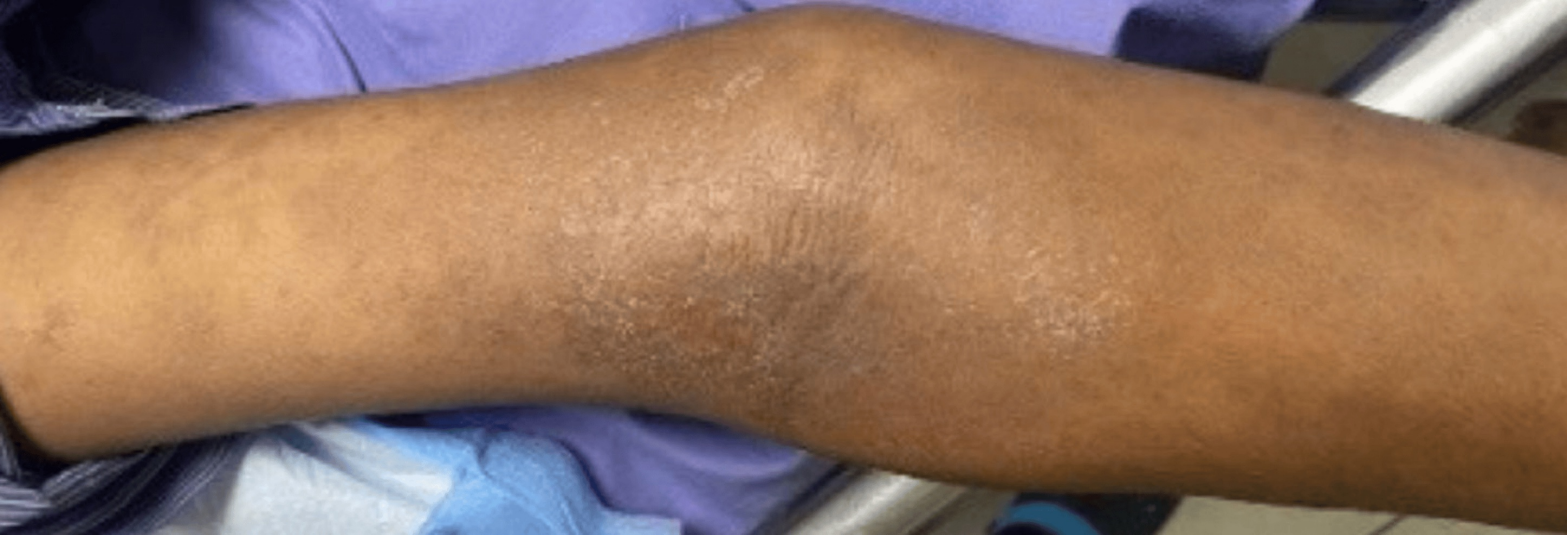
Case 3: Maculopapular rash on the flexor aspect of the forearm.

A 50-year-old male with pontine hemorrhage was on valproate 500 mg once daily. After three weeks, he presented with fever and a maculopapular rash on the upper back, torso, and elbow flexors (Figure 3), accompanied by desquamation, dyspnea, and diarrhea. Laboratory findings showed leukocytosis, eosinophilia, elevated C-reactive protein (CRP), and elevated liver enzymes (5 × ULN). Valproate was discontinued, and intravenous dexamethasone (8 mg daily) was administered for five days. The rashes resolved rapidly by day 3, but sepsis prolonged his hospitalization to 35 days; he was subsequently transferred to another hospital for further care.

Case 4

**Figure 4:**
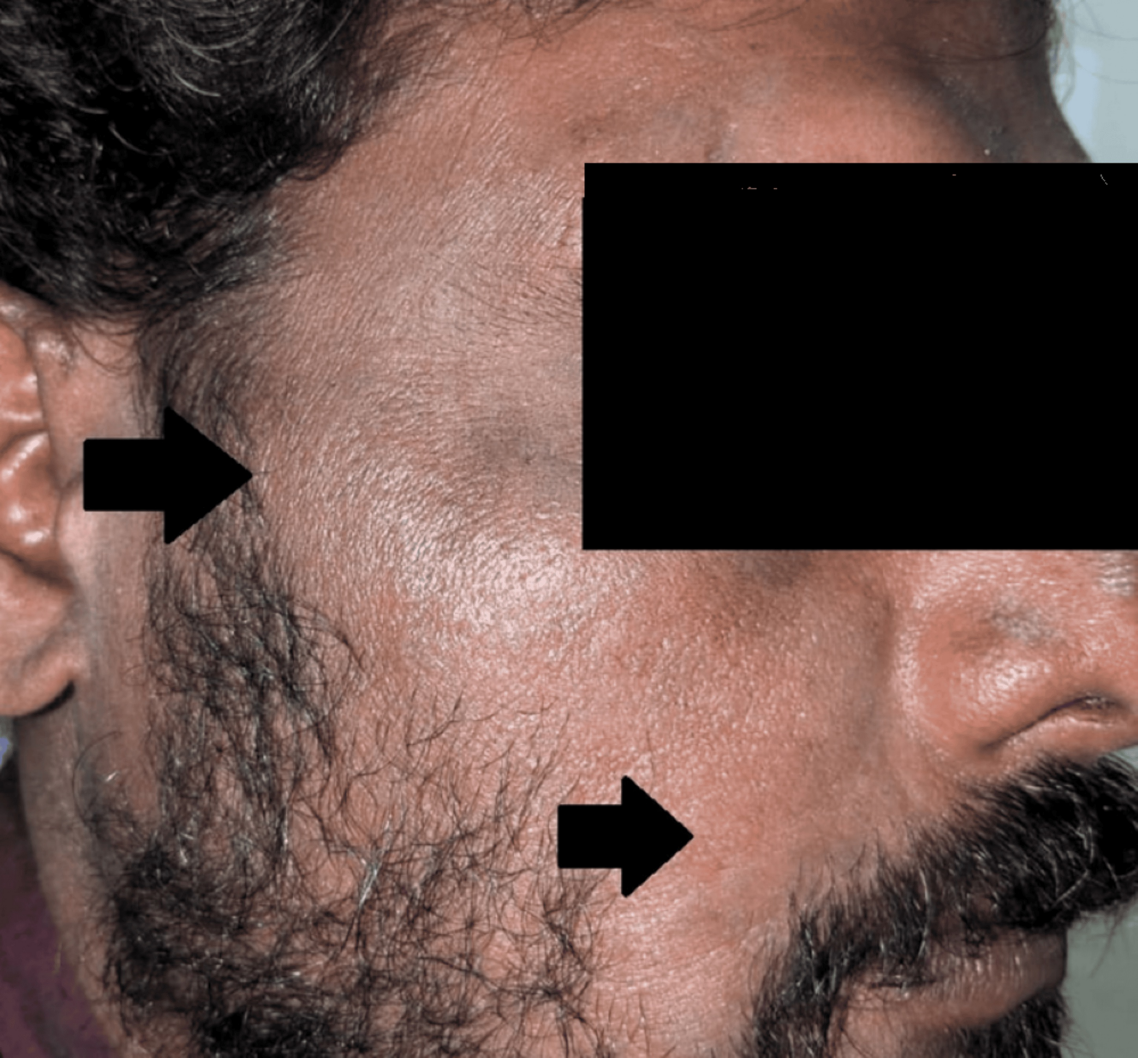
Case 4: Maculopapular rash on the face.

A 40-year-old male was started on phenytoin 200 mg once daily for extradural hemorrhage. Two weeks later, he developed a diffuse maculopapular rash on the face (Figure 4) and trunk, accompanied by pruritus, facial puffiness, fever, nausea, abdominal pain, and bilateral pedal edema. Laboratory results showed marked eosinophilia (21%), lymphocytosis, and elevated liver enzymes (3-5 × ULN). Phenytoin was discontinued, and intravenous dexamethasone (8 mg daily) was administered for five days. The patient subsequently improved and was discharged after 10 days.

Case 5

**Figure 5:**
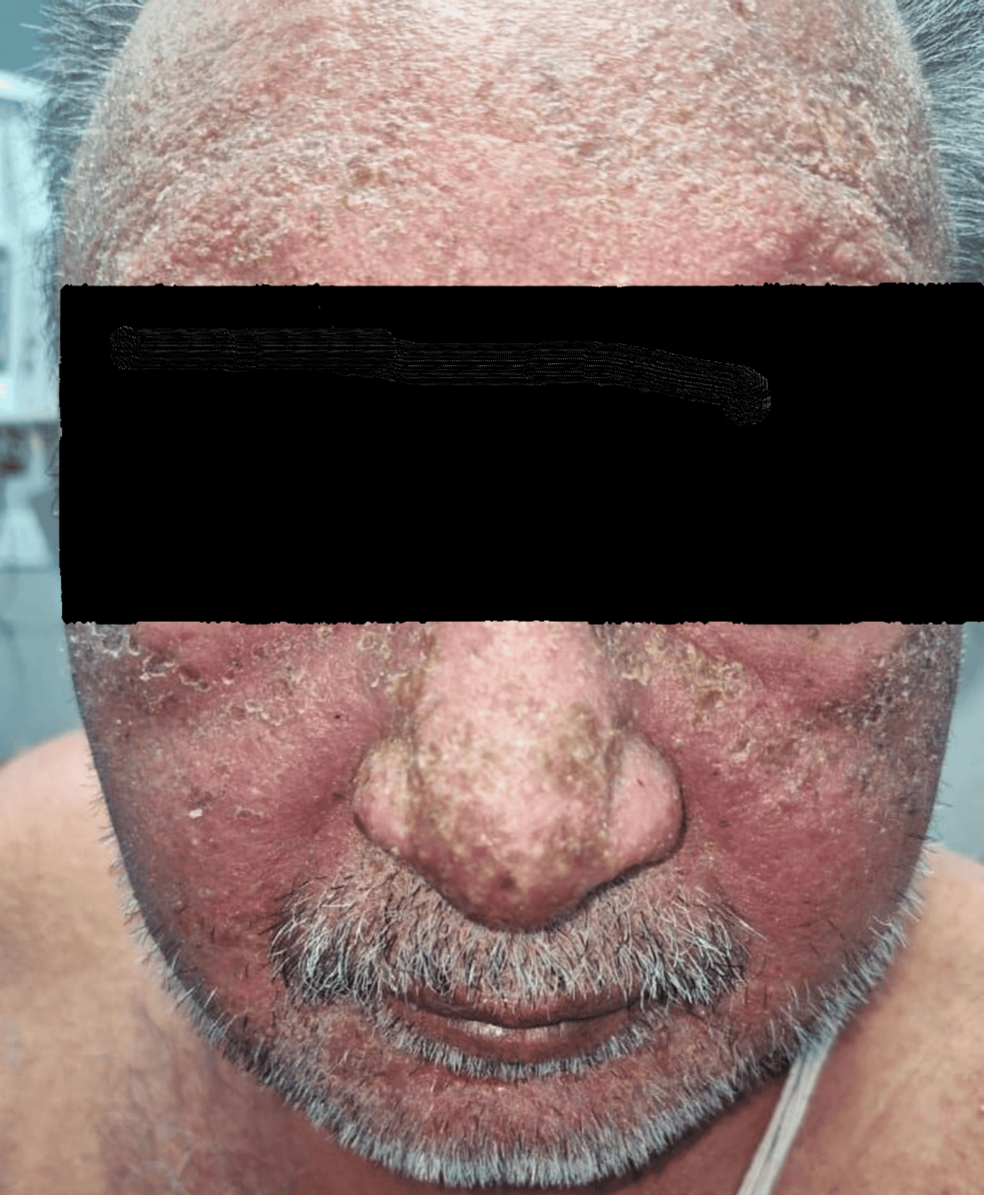
Case 5: Facial swelling with erythema involving the forehead, nose, and malar areas.

A 64-year-old male with hypertension and post-traumatic intracerebral haemorrhage was on phenytoin 200 mg once daily. Three weeks later, he presented with itching, facial and periorbital swelling (Figure 5), erythematous papules with exfoliation on the limbs and trunk, eye watering, nausea, vomiting, and abdominal pain. Laboratory results revealed lymphocytosis, eosinophilia (24%), ALT 2 × ULN, elevated creatinine, and hyponatremia. He was treated with intravenous dexamethasone 8 mg daily for five days. He was discharged after 12 days, and full resolution occurred by 20 days.

Case 6

A 28-year-old female with schizophrenia was on oxcarbazepine 300 mg once daily. She developed diffuse exanthem, axillary lymphadenopathy, fever, abdominal pain, facial/periorbital edema, bilateral pedal edema after 10 days of therapy. Lab results showed eosinophilia (30%), lymphocytosis, elevated bilirubin and elevated liver enzymes (6 × ULN). Oxcarbazepine was stopped. The patient did not respond to intravenous dexamethasone 8 mg even after three days. Subsequently, she received intravenous methylprednisolone 500 mg daily for three days followed by an oral prednisolone tapering for 28 days, with clinical resolution by day 17 and discharged on day 22. Full recovery occurred by day 45.

Case 7

**Figure 6:**
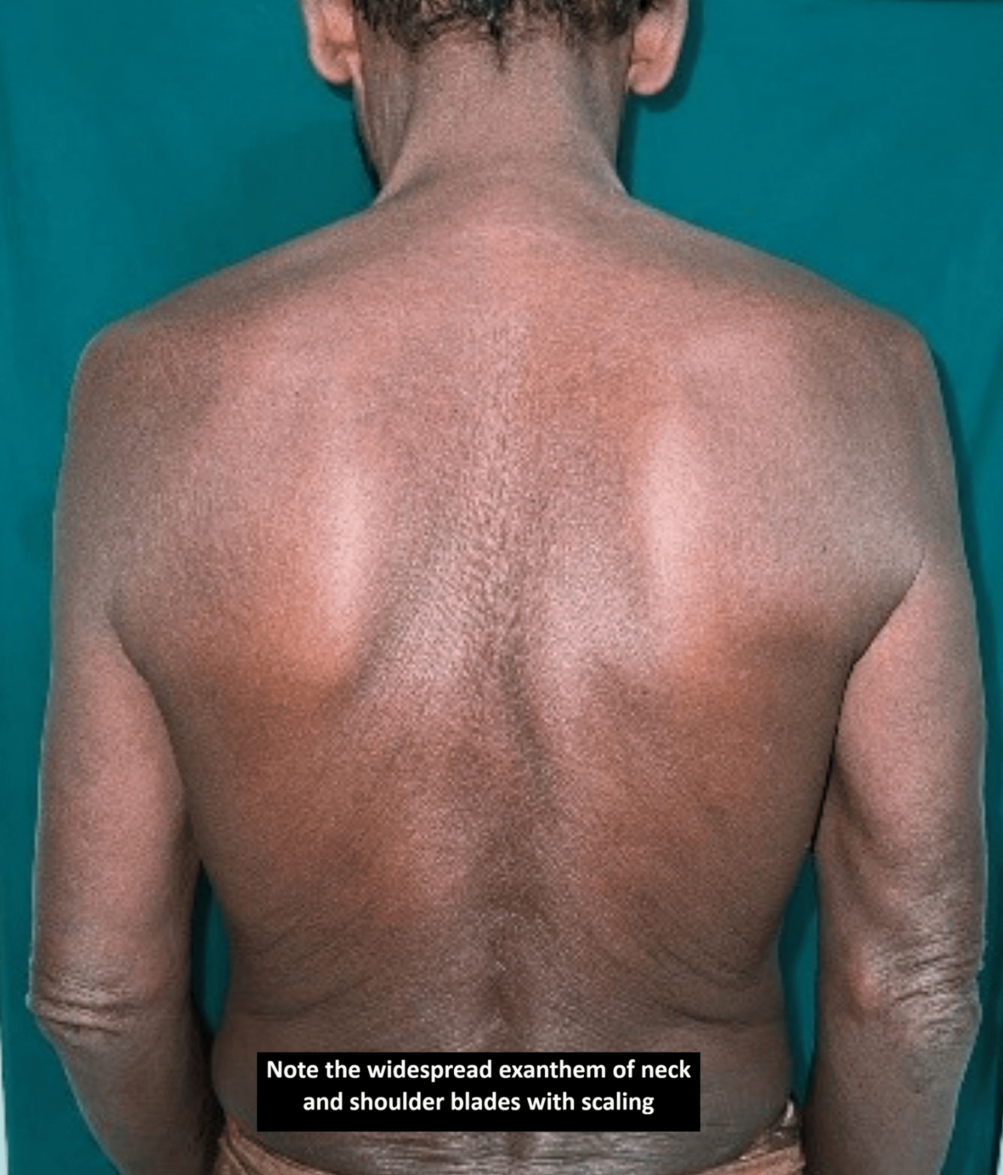
Case 7: Involvement of the back.

**Figure 7:**
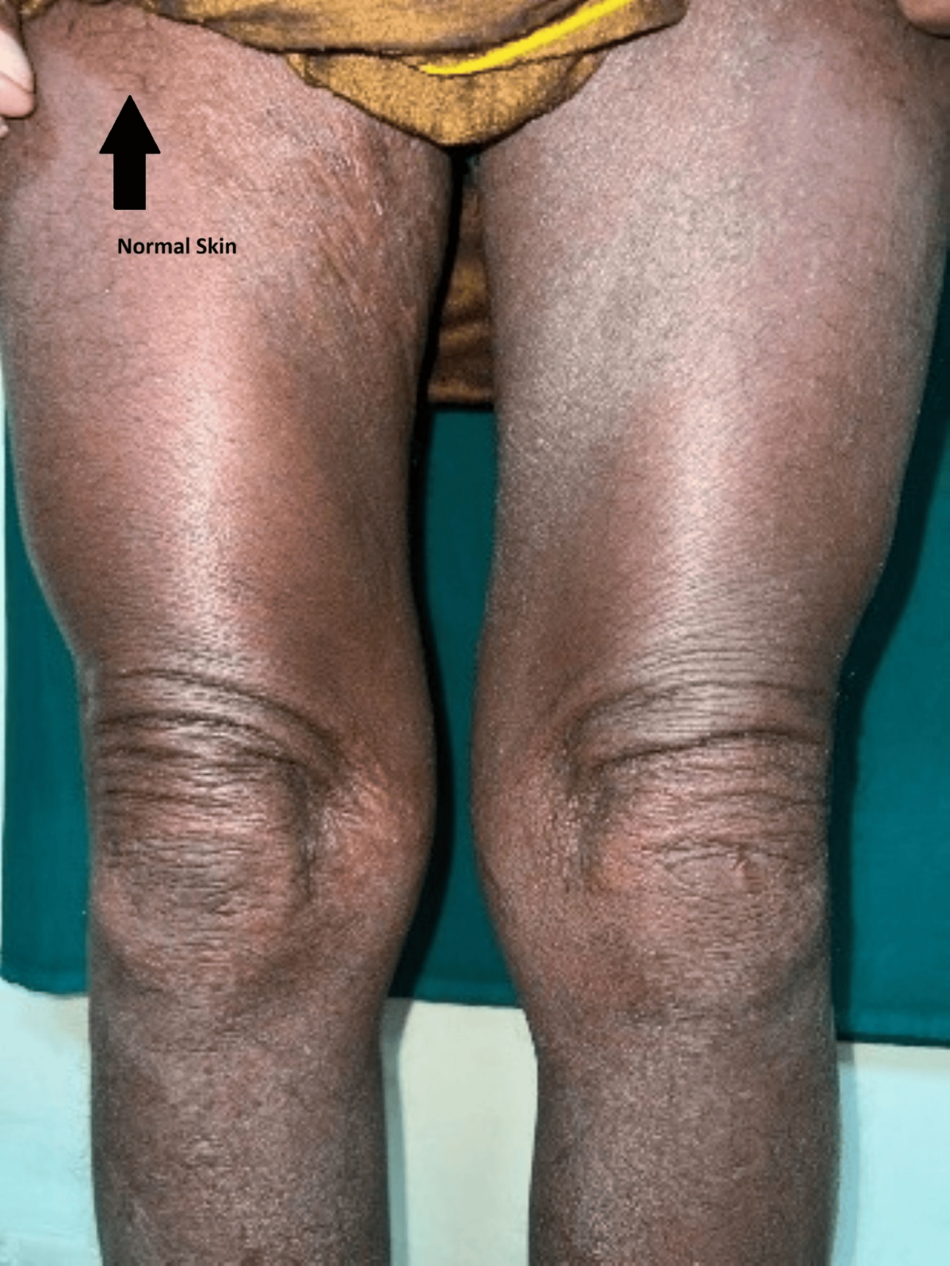
Case 7: Involvement of the limbs with hyperpigmented, scaly macules.

A 45-year-old male with alcohol dependence and cerebral venous thrombosis was on phenytoin 200 mg once daily. Four weeks later, he presented with itching and a diffuse exanthematous macular eruption on the trunk (Figure 6) and lower limbs (Figure 7), accompanied by fever and abdominal pain. Laboratory results showed eosinophilia (27%) and elevated liver enzymes (5 × ULN). The patient was treated with intravenous dexamethasone (8 mg daily) for five days and oral loratadine (10 mg daily) for seven days. Symptoms resolved within three days.

**Figure 8:**
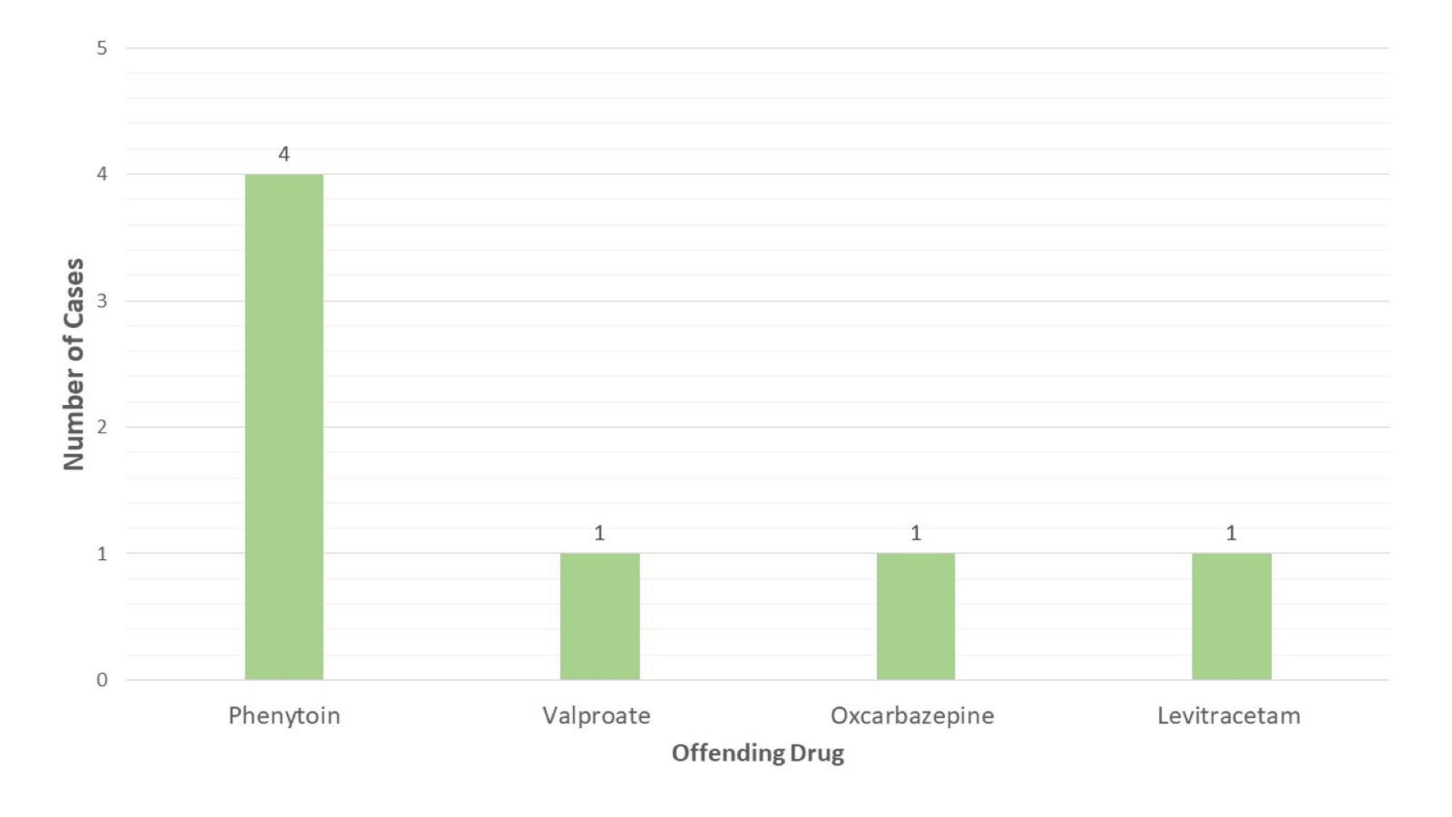
Depiction of the offending drugs.

The offending drugs implicated in the cases described above are shown in Figure 8.

## Discussion

Our case series highlights the variable presentation and diagnostic complexity of AED-induced DRESS, consistent with previous reports [10,21]. The clinical heterogeneity observed ranged from limited cutaneous eruption to multisystem involvement, which underscores that DRESS is a syndrome rather than a single uniform clinical entity. Even though the classic triad of fever, cutaneous eruption, and eosinophilia was frequently present, the timing of onset and the relative severity of organ involvement were variable, which complicates early diagnosis.

All cases showed hepatic involvement, the most commonly affected organ, aligning with existing literature [2,22]. In our cohort, transaminitis ranged from mild to severe (2× to >6× ULN), and one patient developed concurrent renal impairment. Clinically, liver enzyme elevation often preceded other overt systemic complications, indicating that routine early biochemical investigations after initiating AED therapy could help earlier detection and drug withdrawal.

The latency period for developing the symptoms ranged from five to 28 days, reflecting the time needed for reactive metabolite formation and T-cell activation [7,23]. This wide window highlights the fact that monitoring is required not only in the first few weeks following initiation of the drug but for several weeks afterwards. The shorter latencies seen when patients took leftover medication (observed in case 2) illustrate the importance of proper advice and reviewing medication histories.

Phenytoin was the most common culprit, followed by oxcarbazepine, valproate, and levetiracetam. Aromatic AEDs are well-known triggers due to their metabolic activation via CYP 450 enzymes, leading to reactive arene intermediates that act as haptens [24]. However, non-aromatic AEDs like valproate and levetiracetam were also implicated in our series, underscoring that DRESS is not exclusive to aromatic compounds. Cross-reactivity between aromatic and non-aromatic AEDs, possibly due to prior sensitization or shared metabolic pathways, may explain some of these cases [25].

Recent pharmacogenomic insights reinforce the role of HLA alleles in predisposition, particularly for carbamazepine and phenytoin [8]. Although genetic testing was not performed in our patients, existing evidence supports preemptive screening in high-risk populations [26]. Additionally, reactivation of HHV-6, HHV-7, EBV, and cytomegalovirus, a known cofactor in DRESS, was not assessed in our series but remains an important area for future study [9].

Early recognition and prompt withdrawal of the offending drug remain the cornerstone of management. In our cases, we saw subtle rises in eosinophil count and liver enzymes often preceded by the full-blown DRESS, suggesting that routine monitoring within the first 2-12 weeks after AED initiation will help in early diagnosis and management [13,27]. Second, systemic corticosteroids were effective in all of our patients, with most responding to intravenous dexamethasone followed by an oral taper; two patients only required methylprednisolone pulse therapy. This prompt recovery supports the importance of early intervention in treating all patients [28].

Our findings support several measures to reduce morbidity in patients: (1) patient education at the time of AED initiation to report fever or rash immediately to the treating physician; (2) early and regular laboratory monitoring of complete blood count (CBC) and liver function tests (LFTs) during the first weeks of therapy; and (3) reporting of suspected cases to national pharmacovigilance systems to improve signal detection.

In the future, it is necessary to establish prospective multicenter registries that combine clinical phenotyping with genetic and virologic testing and standardized treatment protocols to permit comparative effectiveness research for early diagnosis and treatment. Because of the relative rarity but high morbidity of DRESS, collaborative efforts are necessary to detect a sufficient number of cases for effective analysis.

Limitations

This is a case series, and it includes a small sample from a single center, limiting generalizability. Genetic susceptibility testing and viral reactivation studies were not performed.

## Conclusions

AED-induced DRESS is a severe, multisystem adverse reaction that demands high clinical suspicion and prompt intervention. Phenytoin was the most commonly implicated agent in our case series. Liver involvement was observed in all cases. In the majority of patients, early initiation of systemic corticosteroids led to favorable clinical outcomes. Implementation of structured pharmacovigilance measures, including early warning systems and routine laboratory monitoring, can significantly reduce morbidity.
